# The Efficacy and Safety of Transarterial Chemoembolization Plus Iodine 125 Seed Implantation in the Treatment of Hepatocellular Carcinoma With Oligometastases: A Case Series Reports

**DOI:** 10.3389/fonc.2022.828850

**Published:** 2022-05-17

**Authors:** Weihua Zhang, Linxia Wu, Lei Chen, Yanqiao Ren, Tao Sun, Bo Sun, Licheng Zhu, Yiming Liu, Chuansheng Zheng

**Affiliations:** ^1^Department of Radiology, Union Hospital, Tongji Medical College, Huazhong University of Science and Technology, Wuhan, China; ^2^Department of Interventional Radiology, Union Hospital, Tongji Medical College, Huazhong University of Science and Technology, Wuhan, China

**Keywords:** hepatocellular carcinoma, oligometastasis, transarterial chemoembolization, iodine 125, efficacy, safety

## Abstract

**Background:**

Patients with different primary tumor oligometastases can obtain survival benefits from external radiotherapy. The study was conducted to explore the efficacy and safety of transarterial chemoembolization (TACE) plus iodine 125 seed (TACE-I) implantation for hepatocellular carcinoma (HCC) oligometastases.

**Methods:**

187 patients who received TACE-I in our institution were retrospectively reviewed from January 2014 to December 2018. Thirty-two patients were included in the analysis. The primary endpoints of the study were overall survival (OS) and progression-free survival (PFS). The secondary endpoints of the study were tumor response and PFS of the metastatic sites.

**Results:**

The median OS (mOS) of patients was 18 months, and the median PFS (mPFS) was 7 months. The objective response rate (ORR) and disease control rate (DCR) of patients three months after receiving TACE-I were 34.4% and 71.9%, respectively. The ORR and DCR of patients for metastatic sites were 50% and 81.3%, respectively. The mPFS of patients for metastatic sites was 14 months. The univariable and multivariable regression analyses indicated that the ECOG score was an independent predictor for mOS and mPFS. The number of iodine seeds and ECOG scores were independent predictors for mPFS for metastatic sites. After patients received TACE-I, the most common adverse events were abdominal pain, fever, and appetite. The adverse events of patients were relieved after receiving symptomatic treatments.

**Conclusion:**

Iodine 125 seed implantation may be an effective and safe treatment for patients with hepatocellular carcinoma with oligometastasis, thereby providing a new selective option for these patients.

## Introduction

Liver cancer is one of the most common and fatal cancers (1). There are 905677 new cases of liver cancer worldwide in 2020 (2). Of these patients, approximately 90% are diagnosed with hepatocellular carcinoma (HCC) (3). Radical treatments, such as transplantation, liver resection or ablation, are recommended as the first-line treatments for patients with early HCC because they can improve the 5-year survival rate of patients (4). However, approximately 80% of patients are diagnosed with intermediate to advanced HCC because the symptoms of patients with early HCC are not obvious (5). Previous studies have shown that patients with early HCC have potential tumor metastasis, which is one of the reasons why patients with early-stage tumors still have tumor recurrence after surgery (6). HCC patients with invasion of distant organs are considered to be in an advanced stage (4). For patients with advanced HCC, Atezolizumab plus Bevacizumab therapy is recommended as a strong first-line treatment because the median overall survival of patients after Atezolizumab plus Bevacizumab therapy is 19.2 months, which is longer for patients treated with sorafenib or lenvatinib alone (7–9).

Recently, patients with tumor metastases to three organs with no more than five tumors or HCC metastases to a single organ with no more than 3 tumors were considered that they had tumors oligomestases (10). These patients have not been considered at an advanced stage because their survival times can be extended if they receive suitable treatments (11–15). Oligomestases are considered the transitional state from the intermediate to advanced stage. For these patients, the guideline does not provide exact treatments (4). However, some randomized controlled studies have shown that HCC patients with oligometastases may obtain survival benefits from external radiotherapy (16–19).

The results of external radiotherapy in the treatment of HCC patients with oligometastases are encouraging, but no study has focused internal radiotherapy on treating HCC patients with oligometastases. Iodine 125 seed implantation is an internal radiotherapy. The seeds are implanted into the tumor under the guidance of CT or ultrasound. After being implanted, the seeds emit rays to the tumor cells, which alter the DNA to kill the tumor cells (20, 21). Previous studies have shown that patients with HCC obtain survival benefits from iodine 125 seed implantation, especially when combined with transarterial chemoembolization (TACE) (22–24). However, it remains unknown whether patients with oligometastases obtain survival benefits from TACE plus iodine 125 seed implantation (TACE-I). The present study was conducted to explore the efficacy and safety of TACE-I in the treatment of HCC patients with oligometastases.

## Materials and Methods

### Patient Selection

From January 2014 to December 2018, a total of 187 HCC patients who received TACE-I were retrospectively reviewed. Thirty-two patients were included in the study based on the inclusion criteria and exclusion criteria. The study was conducted in accordance with the Declaration of Helsinki, and it was approved by the Ethics Committee Board of Tongji Medical College, Huazhong University of Science and Technology. Informed consent from the patients was waived by the board because the study was a retrospective study.

**The inclusion criteria were as follows:** (1) patients were diagnosed with HCC according to the EASL guidelines by imaging or biopsy (4); (2) patients received TACE-I treatment; (3) patients without portal vein tumor thrombus.

**Figure 1:**
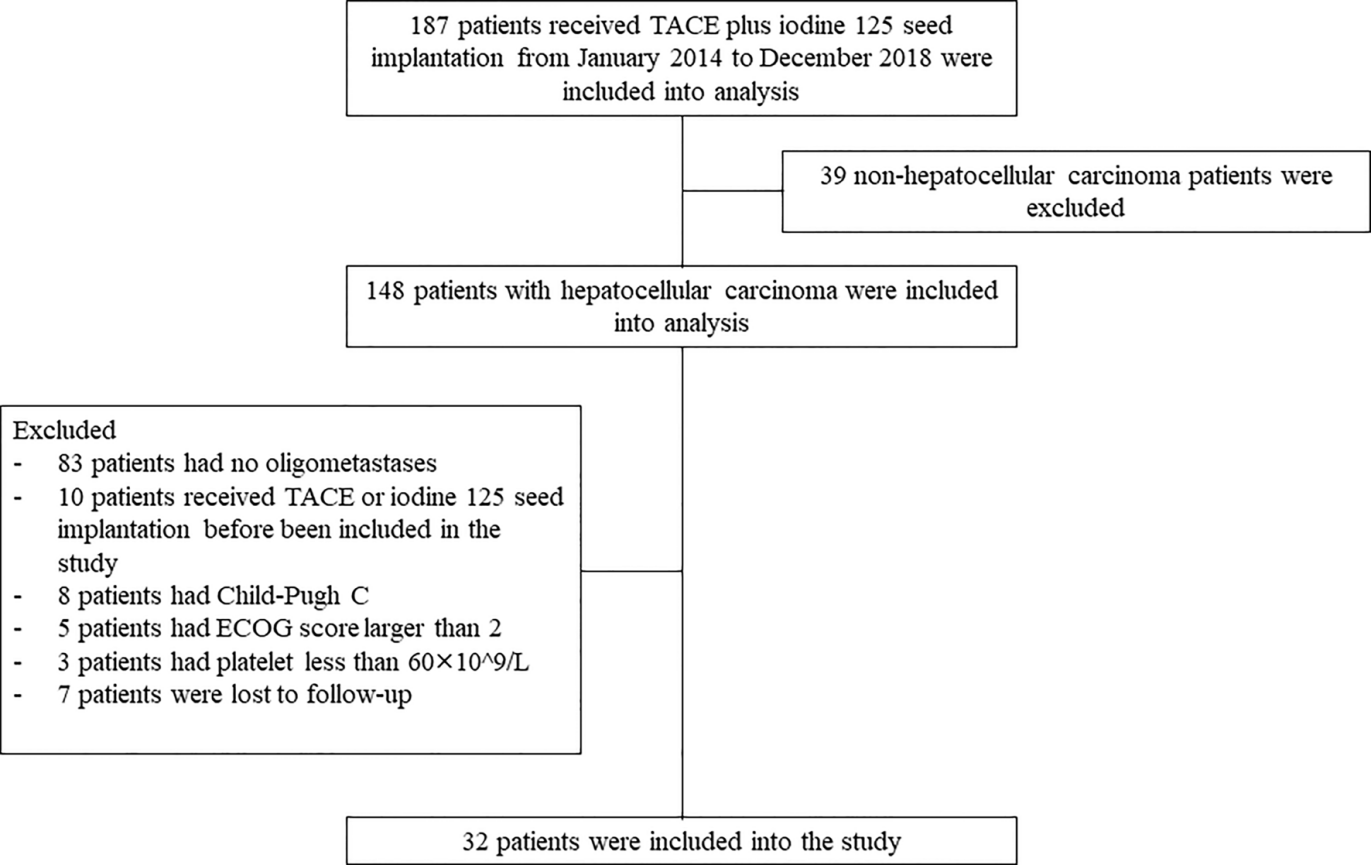
The flowchart of patient selection.

**The exclusion criteria were as follows:** (1) patients with no oligometastases; (2) patients received TACE or iodine 125 seed implantation before included in the study; (3) patients with Child-pugh C; (4) patients with ECOG score larger than 2; (5) patients with platelet count less than 60^×^10^^^9/L; and (6) patients lost to follow-up (**Figure 1**).

### TACE Procedure

TACE was performed for the treatment of intrahepatic tumors. First, a 5F catheter was introduced to the femoral artery by the Seldinger method, and it was then placed into the celiac trunk. Celiac angiography was conducted to relocate the tumor. A 5F catheter or 3F microcatheter was then introduced into the tumor feeding artery. Finally, lipiodol (10-20 ml), epirubicin (20-40 mg) and subsequent absorbable gelatin sponge particles with diameters ranging from 350 to 560 μm were injected into the tumor feeding artery.

### Iodine 125 Seed Implantation Procedure

**Figure 2:**
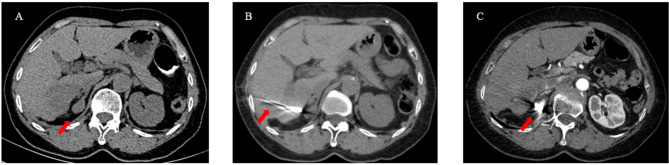
A 63-year-old woman with HCC oligometastases. **(A)** The CT scan showed HCC oligometastasis (red arrow) in the right adrenal gland; **(B)** Under the guidance of CT, iodine 125 seeds were implanted into the metastatic by a 18G needle and turntable implantation gun (red arrow); **(C)** The CT scan showed that the Iodine 125 seeds were evenly distributed (red arrow).

Three to seven days after the TACE protocol, iodine 125 seed implantation was performed for the treatment of extrahepatic tumors. Iodine 125 seeds, which were enclosed in NiTinol capsules, were purchased from the Institute of Atomic Energy, Beijing. Before the implantation of iodine 125 seeds, patients received a computed tomography (CT) scan, and the images were then transmitted to the treatment-planning system (TPS). The number of iodine 125 seeds was determined by TPS in accordance with the minimum peripheral dose (90–165 Gy). Under the guidance of CT and ultrasound, the iodine seeds were implanted into the tumor at intervals of 1 to 1.5 cm by 18 G needles and a turntable implantation gun (XinKe Pharmaceutical Ltd., Shanghai, China) (**Figure 2**).

### Study Endpoints

The primary endpoints of the study were overall survival (OS) and progression-free survival (PFS). The secondary endpoints were PFS of the metastatic sites and disease control rate (DCR). OS was defined as the interval from the time of initial TACE to the time of patient death or the end of the study. PFS was defined as the interval from the time patients received the first treatment of iodine 125 seed implantation to the time of overall tumor progression or the end of the study. The PFS of the metastatic sites was defined as the interval from the time of the initial iodine 125 seed implantation to the time of metastatic site progression or the end of the study. The DCR was defined as the ratio of patients with tumor complete response (CR), partial response (PR) or stable disease (SD) to all patients. The ORR was defined as the ratio of patients with tumor complete response (CR) or partial response (PR) to all patients. PFS and DCR were evaluated based on the modified Respond Evaluation Criteria in Solid Tumors (mRECIST) (25).

### Follow Up

All patients who received TACE or iodine 125 seed implantation in the institution were followed. Patients were diagnosed with HCC oligometastases based on PET-CT, enhanced CT, enhanced MRI or whole body bone imaging. In the study, patients were required to undergo CT, MRI, ultrasound or laboratory examination every month after the initial iodine 125 seed implantation and then once every two or three months. The tumor response was evaluated by two radiologists (one with 15 years of experience and another with 8 years of experience) and one interventional radiologist (with 32 years of experience). The patients were recommended to receive another TACE or iodine 125 seed implantation if the imaging of the patients showed primary tumors, metastatic site progression or remaining tumors. The end time of the study was June 2021.

### Statistical Analysis

The continuous variables were compared by the paired sample t test. The Kaplan–Meier method was used to plot the survival curves. Cox regression analysis was used to evaluate the variables that may influence the outcomes. The variables with P less than 0.05 in the univariable regression analysis were included in the multivariable regression analysis. A nested graph was used to show the changes in liver function before TACE-I and after TACE-I. All P values were two-tailed, and a P value less than 0.05 was considered statistically significant. All statistical analyses were conducted by SPSS. 26.0 (IBM Corp., Armonk, NY, USA).

## Results

### Patient Characteristics

A total of 187 patients received TACE plus iodine 125 seed implantation from January 2014 to December 2018. Based on the inclusion criteria and exclusion criteria, 32 patients were included in the study. Among them, 5 patients received sorafenib and 2 patients received apatinib before been included into the study. 26 patients had single organ metastases, and 6 patients had multiple organ metastases. Single primary tumor was present in 14 patients, and multiple tumors were present in 18 patients. Eight patients received TACE once, and 24 patients received TACE multiple times. 25 patients received one iodine 125 seed implantation, and 7 patients received multiple iodine seed implantations (**Table 1**).

**Table 1:** The baseline characteristics of patients with TACE-I.

Characteristics	TACE+Iodine 125 seed implantation
**Age (years)**	57.5 ± 12.5
**ALT (U/L)**	44 ± 32.5
**AST (U/L)**	43.8 ± 30.3
Leukocyte (*10^9^/L)	5.1 ± 2.4
Neutrophils (*10^9^/L)	4.3 ± 0.5
Lymphocytes (*10^9^/L)	1.3 ± 0.6
Erythrocyte (*10^12^/L)	4.3 ± 0.5
Platelet (*10^9^/L)	146.3 ± 61.5
**Primary tumor size (cm)**	5.8 ± 2.8
**Metastatic tumor size (cm)**	3 ± 1.5
**Number of iodine seeds**	29.4 ± 20.8
**Gender**	
Male	27
Female	5
**HBV**	
Yes	20
No	12
**Cirrhosis**	
Yes	24
No	8
**Primary tumor number**	
1	15
≥2	17
**Metastatic tumor number**	
1	15
2	15
3	2
**Metastatic sites**	
Single organ metastases	26
Multiple organs metastases	6
**AFP level**	
>200	15
<200	17
**TACE session**	
1	8
≥2	24
**Iodine 125 seeds implantation session**	
1	25
≥2	7
**Child-Pugh**	
A	23
B	9
**ECOG**	
0	9
1	13
2	10

### Survival Outcomes and Tumor Response

**Figure 3:**
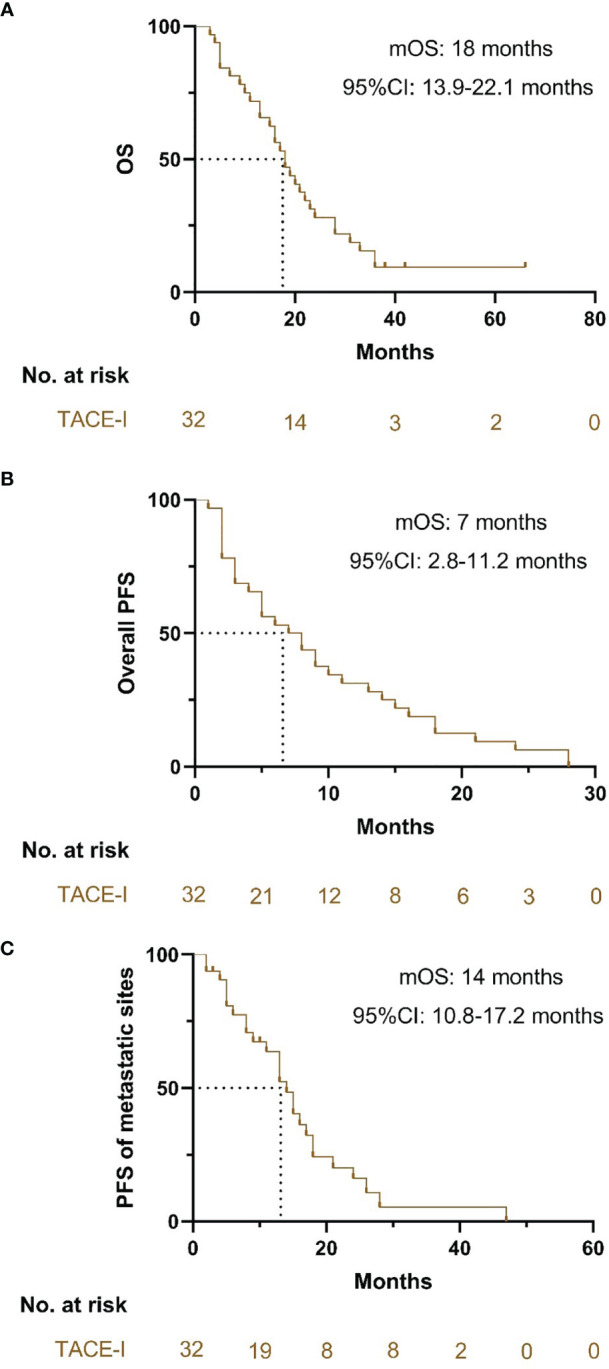
Kaplan-Meier curves for OS, PFS and PFS of metastatic sites. **(A)** Kaplan-Meier curves for OS; **(B)** Kaplan-Meier curves for PFS; **(C)** Kaplan-Meier curves for PFS of the metastatic sites.

The median OS of patients with TACE-I was 18 months (95% CI: 13.9-22.1 months) (**Figure 3A**). The median PFS of patients was 7 months (95% CI: 2.8-11.2 months) (**Figure 3B**), and the median PFS of the metastatic sites was 14 months (95% CI: 10.8-17.2 months) (**Figure 3C**). The overall DCR three months after the treatment was 71.9%, and the DCR of metastatic sites was 81.3% (**Table 2**).

**Table 2:** The tumor response for overall tumor burden and metastatic sites.

Tumor response	Overall	Metastatic sites
**Complete response**	2 (6.3%)	4 (12.5%)
**Partial response**	9 (28.1%)	12 (37.5%)
**Stable disease**	12 (37.5%)	10 (31.3%)
**Progressive disease**	9 (28.1%)	6 (18.7%)
**ORR**	34.4%	50%
**DCR**	71.9%	81.3%

### Predictors for OS PFS and PFS of Metastatic Sites

In the Cox regression analysis for OS, the univariable regression analysis showed that primary tumor size (HR: 1.223, 95% CI: 1.058-1.413, P=0.006) and ECOG (1 vs. 0: HR: 12.857, 95% CI: 2.664-62.043, P=0.001; 2 vs. 0: HR: 70.286, 95% CI: 11.547-427.833, P<0.001) were predictors for OS. In the multivariable regression analysis, only ECOG (1 vs. 0: HR: 13.024, 95% CI: 2.687-63.114, P=0.001) was an independent predictor for OS (**Table 3**).

**Table 3:** Univariable regression analysis and multivariable regression analysis for OS.

Characteristics	Univariable analysis		Multivariable analysis	
	HR (95%CI)	P value	HR (95%CI)	P value
**Age (years)**	0.979 (0.950,1.008)	0.150		
**ALT (U/L)**	1.010 (0.999,1.021)	0.074		
**AST (U/L)**	0.999 (0.988,1.009)	0.798		
**Leukocyte**	1.182 (0.973,1.436)	0.093		
**Neutrophils**	1.289 (0.989,1.679)	0.060		
**Lymphocytes**	0.823 (0.438,1.546)	0.544		
**Erythrocyte (◊10^12^/L)**	1.590 (0.692,3.649)	0.274		
**Platelet**	1.006 (0.999,1.013)	0.075		
**Primary tumor size (cm)**	1.223 (1.058,1.413)	0.006	0.973 (0.812,1.166)	0.768
**Metastatic tumor size (cm)**	1.218 (0.988,1.502)	0.065		
**Number of iodine seeds**	1.012 (0.996,1.028)	0.146		
**Gender**				
Male	Ref			
Female	0.576 (0.199,1.668)	0.309		
**HBV**				
Yes	Ref			
No	0.890 (0.413,1.919)	0.766		
**Cirrhosis**				
Yes	Ref			
No	0.764 (0.309,1.885)	0.559		
**Primary tumor number**				
1	Ref			
≥2	1.370 (0.635,2.958)	0.423		
**Metastatic tumor number**				
1	Ref			
2	0.795 (0.371,1.702)	0.555		
3	0.893 (0.198,4.033)	0.883		
**Metastatic sites**				
Single organ metastases	Ref			
Multiple organs metastases	0.649 (0.258,1.634)	0.359		
**AFP level**				
>200	Ref			
<200				
**TACE session**				
1	Ref			
≥2	0.851 (0.408,1.776)	0.668		
**Iodine 125 seeds implantation session**				
1	Ref			
≥2	0.598 (0.240,1.490)	0.270		
**Child-Pugh**				
A	Ref			
B	1.417 (0.643,3.121)	0.387		
**ECOG**				
0	Ref		Ref	
1	12.857 (2.664,62.043)	0.001	13.024 (2.687,63.114)	0.001
2	70.286 (11.547,427.833)	<0.001	49.989 (7.630,327.484)	<0.001

In the Cox regression analysis for PFS, the univariable regression analysis showed that age (HR: 0.969, 95% CI: 0.939-0.999, P=0.046), metastatic sites (HR: 0.366, 95% CI: 0.136-0.985, P=0.047) and ECOG (2 vs. 0: HR: 10.693, 95% CI: 3.135-36.466, P<0.001) were predictors for PFS. In the multivariable regression analysis, the results showed that ECOG (2 vs. 0: HR: 9.038, 95% CI: 2.300-35.521, P=0.002) was an independent predictor for PFS. (**Table 4**)

**Table 4:** Univariable regression analysis and multivariable regression analysis for PFS.

Characteristics	Univariable analysis		Multivariable analysis	
	HR (95%CI)	P value	HR (95%CI)	P value
**Age (years)**	0.969 (0.939,0.999)	0.046	0.998 (0.964,1.033)	0.144
**ALT (U/L)**	1.004 (0.994,1.014)	0.437		
**AST (U/L)**	0.998 (0.988,1.008)	0.713		
**Leukocyte**	1.126 (0.935,1.356)	0.210		
**Neutrophils**	1.002 (0.996,1.009)	0.506		
**Lymphocytes**	0.797 (0.436,1.457)	0.461		
**Erythrocyte (◊10^12^/L)**	1.205 (0.550,2.639)	0.641		
**Platelet**	1.002 (0.996,1.009)	0.506		
**Primary tumor size (cm)**	1.174 (0.997,1.383)	0.054		
**Metastatic tumor size (cm)**	1.136 (0.879,1.470)	0.330		
**Number of iodine seeds**	1.015 (0.997,1.034)	0.108		
**Gender**				
Male	Ref			
Female	0.385 (0.131,1.134)	0.083		
**HBV**				
Yes	Ref			
No	0.721 (0.340,1.530)	0.394		
**Cirrhosis**				
Yes	Ref			
No	0.736 (0.311,1.741)	0.485		
**Primary tumor number**				
1	Ref			
≥2	1.597 (0.753,3.386)	0.222		
**Metastatic tumor number**				
1	Ref			
2	1.141 (0.538,2.419)	0.732		
3	1.487 (0.328,6.741)	0.607		
**Metastatic sites**				
Single organ metastases	Ref		Ref	
Multiple organs metastases	0.366 (0.136,0.985)	0.047	0.458 (0.160,1.306)	0.144
**AFP level**				
>200	Ref			
<200	0.694 (0.333,1.445)	0.329		
**TACE session**				
1	Ref			
≥2	0.500 (0.217,1.152)	0.104		
**Iodine 125 seeds implantation session**				
1	Ref			
≥2	0.428 (0.166,1.099)	0.078		
**Child-Pugh**				
A	Ref			
B	1.328 (0.605,2.915)	0.479		
**ECOG**				
0	Ref		Ref	
1	1.315 (0.522,3.317)	0.561	1.361 (0.527,3.510)	0.524
2	10.693 (3.135,36.466)	<0.001	9.038 (2.300,35.521)	0.002

In the Cox regression analysis for PFS of the metastatic sites, the univariable regression analysis showed that age (HR: 0.953, 95%CI: 0.918-0.989, P=0.011), number of iodine 125 seeds (HR: 1.023, 95%CI: 1.002-1.044, P=0.035) and ECOG (2 vs. 0: HR: 8.214, 95%CI: 2.152-31.345, P=0.002) were independent predictors for PFS of the metastatic sites. In the multivariable regression analysis, the results showed that the number of iodine 125 seeds (HR: 1.042, 95% CI: 1.014-1.071, P=0.004) and ECOG (2 vs. 0: HR: 15.365, 95% CI: 3.137-75.272, P=0.001) were independent predictors for PFS of metastatic sites (**Table 5**).

**Table 5:** Univariable regression analysis and multivariable regression analysis for PFS of metastatic sites.

Characteristics	Univariable analysis		Multivariable analysis	
	HR (95%CI)	P value	HR (95%CI)	P value
**Age (years)**	0.953 (0.918,0.989)	0.011	0.970 (0.938,1.004)	0.082
**ALT (U/L)**	1.006 (0.995,1.018)	0.273		
**AST (U/L)**	0.999 (0.988,1.010)	0.851		
**Leukocyte**	0.979 (0.771,1.243)	0.860		
**Neutrophils**	0.948 (0.659,1.362)	0.772		
**Lymphocytes**	0.715 (0.336,1.521)	0.383		
**Erythrocyte (◊10^12^/L)**	1.010 (0.436,2.337)	0.982		
**Platelet**	1.003 (0.996,1.010)	0.409		
**Primary tumor size (cm)**	1.126 (0.936,1.354)	0.207		
**Metastatic tumor size (cm)**	1.151 (0.863,1.534)	0.338		
**Number of iodine 125 seeds**	1.023 (1.002,1.044)	0.035	1.042 (1.014,1.071)	0.004
**Gender**				
Male	Ref			
Female	0.646 (0.236,1.772)	0.396		
**HBV**				
Yes	Ref			
No	1.431 (0.645,3.176)	0.378		
**Cirrhosis**				
Yes	Ref			
No	0.772 (0.299,1.994)	0.593		
**Primary tumor number**				
1	Ref			
≥2	1.980 (0.840,4.663)	0.118		
**Metastatic tumor number**				
1	Ref			
2	1.134 (0.487,2.642)	0.771		
3	3.353 (0.697,16.118)	0.131		
**Metastatic sites**				
Single organ metastases	Ref			
Multiple organs metastases	0.563 (0.204,1.550)	0.266		
**AFP level**				
>200	Ref			
<200	0.712 (0.323,.1.570)	0.400		
**TACE session**				
1	Ref			
≥2	0.512 (0.197,1.330)			
**Iodine 125 seeds implantation session**				
1	Ref			
≥2	0.377 (0.126,1.123)	0.080		
**Child-Pugh**				
A	Ref			
B	1.315 (0.537,3.221)	0.549		
**ECOG**				
0	Ref		Ref	
1	1.825 (0.644,5.166)	0.258	1.282 (0.443,3.707)	0.647
2	8.214 (2.152,31.345)	0.002	15.365 (3.137,75.272)	0.001

### Change of Liver Function and Blood Index

**Figure 4:**
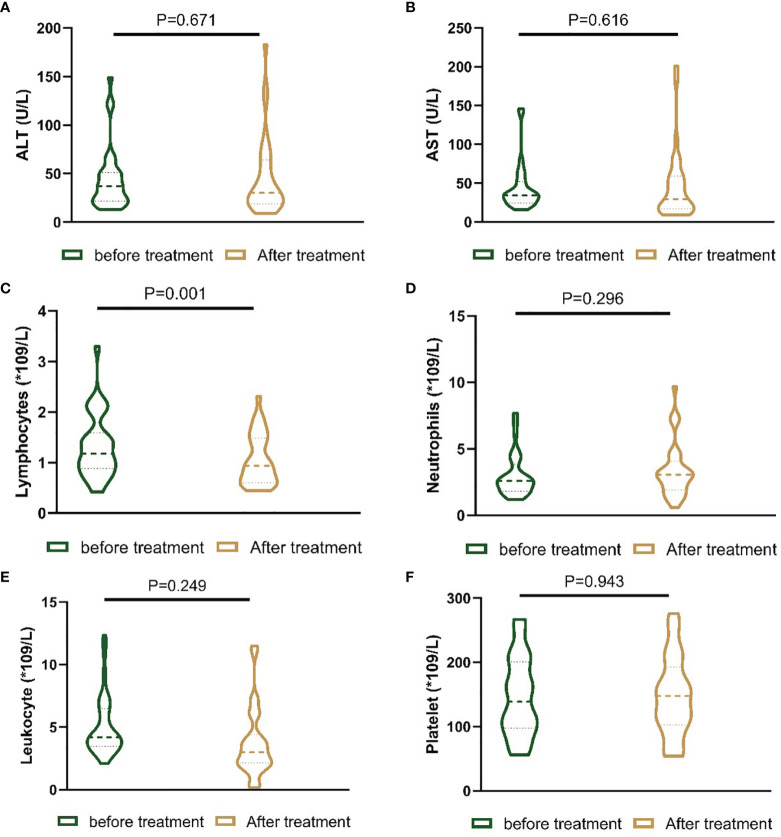
Nested figures for changing of liver function and blood index of patients before the treatment and one month after the treatment. **(A)** Change of ALT; **(B)** change of AST; **(C)** change of lymphocytes; **(D)** change of neutrophils; **(E)** change of leukocyte; **(F)** change of platelet.

In the present study, the liver function and blood index before treatment and one month after treatment were evaluated. There was no statistically significant difference in alanine aminotransferase (ALT), aspartate aminotransferase (AST), leukocyte count, neutrophil count or platelet count. However, the lymphocyte count in the patients one month after receiving the treatments was lower than that in the patients before receiving the treatments (**Figure 4**).

### Adverse Event Analyses

In the present study, nine adverse events of patients after receiving the treatments were reported. The most common adverse event was postembolism syndrome, including fever (53%), abdominal pain (75%), nausea (56.3%), vomiting (21.9%) and poor appetite (78.1%). There were 4 patients with headache, 5 patients with leukopenia, 3 patients with bleeding and 2 patients with radiation pneumonia. For grade III or IV adverse events, 2 patients had severe fever, 5 patients had severe abdominal pain, 1 patient had severe nausea, 1 patient had severe vomiting and 3 patients had a severely poor appetite. The adverse events were alleviated after the patients received symptomatic treatments (**Table 6**).

**Table 6:** Adverse events of patients after receiving the treatments.

Adverse events	All grades (N, %)	III or IV grades (N, %)
**Postembolism syndrome**		
Fever	17 (53.1)	2 (6.3)
Abdominal pain	24 (75)	5 (15.6)
Headache	4 (12.5)	0 (0)
Nausea	18 (56.3)	1 (3.1)
Vomit	7 (21.9)	1 (3.1)
Poor appetite	25 (78.1)	3 (9.4)
**Iodine 125 seeds implantation related adverse events**		
Leukopenia	5 (15.6)	0 (0)
Bleeding	3 (9.4)	0 (0)
Radiation pneumonia	2 (6.3)	0 (0)

## Discussion

Iodine 125 seeds alone or combined with TACE have been used in the treatment of different stages of HCC with encouraging results (22, 23). Patients with oligometastases are considered to have a transition state between an intermediate stage and an advanced stage. High-quality studies have demonstrated that these patients may obtain survival benefits from external radiotherapy (12, 17, 18). However, it remains unclear whether these patients obtain survival benefits from internal radiotherapy. The present study was conducted to explore the efficacy of TACE plus iodine 125 seed implantation in patients with HCC oligometastases.

The main findings of the present study were that patients with HCC oligometastases obtain survival benefits from TACE-I and can tolerate the treatments. In the present study, the mOS of patients was 18 months, and the mPFS of patients was 7 months. In addition, the mPFS of metastatic sites was 14 months. The mOS and mPFS of the patients in the present study were longer than the mOS and mPFS of patients with advanced HCC in previous high-quality studies. Recently, atezolizumab plus bevacizumab has been recommended as a strong first-line treatment for patients with advanced HCC instead of sorafenib and lenvatinib (weak first-line treatment) because the mOS of patients with advanced HCC who received the combination treatment was 19.2 months and the mPFS was 6.8 months (3, 7, 9). The mOS and mPFS of the present study were similar to the results from the IMbrave 150 study (7). However, the patients included in the present study and the IMbrave study were different. It remains unclear whether patients with HCC oligometastases obtain survival benefits from immunotherapy plus tyrosine kinase inhibitors, and more studies are required to explore this treatment. Chen JB et al. studied 40 HCC patients with pulmonary oligometastases who received sorafenib plus regional therapies (radiofrequency ablation, iodine 125 seeds and resection) (26); they reported that the mOS was 18.37 months, which was similar to the mOS in the present study, and the median time to progression was shorter than the mPFS in the present study. The differences may be due to the inclusion of patients with macrovascular invasion in their study, which may influence the tumor response. In the present study, the overall ORR and DCR were higher than those in previous studies (14, 27, 28), indicating that the combination treatment may limit tumor progression. Additionally, we evaluated the metastatic tumor responses after the treatment. The mPFS, ORR and DCR of metastatic sites were longer than the overall mPFS in the present study and the mPFS in previous studies, demonstrated that iodine 125 seeds control metastatic tumors.

After exclusion of potential variables that might influence the results, the Cox regression analysis showed that higher ECOG scores were independent worse predictors for OS, PFS and PFS of metastatic sites. Thus, for patients with HCC oligometastases, lower ECOG scores of patients with HCC oligometastases before they receive TACE-I might result in more survival benefits.

In the evaluation of the safety of patients included in the study, a total of nine adverse events were reported. Most of the patients had mild adverse events, but some patients were reported to have severe adverse events (grades III or IV). The adverse events of patients were alleviated after they received symptomatic treatments. Moreover, the dynamic changes in liver functions and blood indices were evaluated. Only lymphocyte counts of patients one month after the treatment were significantly lower than those before the treatment. For future follow-up, the lymphocyte count was within the normal range. In the study, 2 patients had radiation pneumonia after they receiving iodine 125 seeds implantation. It might be that there were 5 patients with HCC metastasized to lung. And the iodine 125 seeds were implanted in the tumors located in lung, which lead to radiation pneumonia. The results of the adverse event analyses showed that patients with HCC oligometastases may tolerate TACE-I.

There present study had several limitations. First, the study was a retrospective study, which led to inevitable selection bias. Second, the sample size was small, which may lead to a low level of evidence. Thus, future prospective studies or large sample retrospective studies are needed to confirm the conclusion of the present study.

## Conclusion

TACE-I may be effective and safe in the treatment of patients with hepatocellular carcinoma oligometastases, thereby providing new evidence for clinics to select suitable treatment for these patients.

## Data Availability Statement

The raw data supporting the conclusions of this article will be made available by the authors, without undue reservation.

## Ethics Statement

The studies involving human participants were reviewed and approved by Ethics Committee Board of Tongji Medical College, Huazhong University of Science and Technology. Written informed consent for participation was not required for this study in accordance with the national legislation and the institutional requirements.

## Author Contributions

WZ, LW, and LC collected the patients’ data. LC, LW, and WZ drafted the manuscript. CZ, YL, BS, and TS revised the manuscript. WZ, LC, LZ, and YR analyzed and interpreted the data. LW and WZ made substantial contributions to the conception of the work. YL and CZ made substantial contributions to the design of the work and have revised the manuscript substantively. All authors read and approved the final manuscript.

## Funding

This study was supported by National Natural Science Foundation of China (No. 81873919 and 81801810).

## Conflict of Interest

The authors declare that the research was conducted in the absence of any commercial or financial relationships that could be construed as a potential conflict of interest.

## Publisher’s Note

All claims expressed in this article are solely those of the authors and do not necessarily represent those of their affiliated organizations, or those of the publisher, the editors and the reviewers. Any product that may be evaluated in this article, or claim that may be made by its manufacturer, is not guaranteed or endorsed by the publisher.

## Abbreviations

HCC, Hepatocellular Carcinoma; TACE-I, Transarterial chemoembolization plus iodine 125 seed implantation; OS, Overall Survival; PFS, Progression-free Survival; ORR, Objective Response Rate; DCR, Disease Control Rate; ECOG, Eastern Cooperative Oncology Group; mRECIST, modified Respond Evaluation Criteria in Solid Tumors; EASL, European Association for the Study of the Liver.
